# Dropping the urine culture: sustained CAUTI reduction using a UTI order panel

**DOI:** 10.1017/ice.2025.2

**Published:** 2025-04

**Authors:** Cristina Torres, Elizabeth Lyden, Gayle Gillett, Mark E. Rupp, Trevor C. Van Schooneveld

**Affiliations:** 1 Division of Infectious Diseases, University of Nebraska Medical Center, Omaha, NE, USA; 2 College of Public Health, University of Nebraska Medical Center, Omaha, NE, USA; 3 Department of Infection Control and Epidemiology, Nebraska Medicine, Omaha, NE, USA

## Abstract

**Objective:**

We introduced a urinary tract infection (UTI) panel requiring symptom documentation and identification of special populations linked to reflex urine culturing and evaluated its impact on catheter-associated UTI (CAUTI) including during the COVID-19 pandemic.

**Design:**

Quasi-experimental encompassing 3 periods: pre-panel (January 2014–March 2015), post-panel (April 2015–March 2020), and post-panel COVID (April 2020–June 2022).

**Setting/participants:**

Tertiary care center inpatients.

**Methods:**

Poisson regression and interrupted time series (ITS) analysis evaluated changes in catheter days (CD), urine cultures (UC), and CAUTI measured by 1,000 CD and patient days (PD). National Health Safety Network standardized infection ratio (SIR) and standardized utilization ratio (SUR) data were analyzed.

**Results:**

UC per 1,000 PD decreased after implementation (pre-panel 36.9 vs 16.6 post-panel vs 14.4 post-panel COVID, all *P* < .001). CD declined pre-panel versus post-panel (RR 0.37, *P* < .001) but not from post-panel to post-panel COVID (RR 0.94, *P* = .88). UTI panel implementation was associated with a 40% decrease in CAUTI rates per 1,000 CD (*P* < .001). During the COVID-19 pandemic (post-panel COVID), a nonsignificant increase of 13% (*P* = .61) in CAUTI was noted but remained 32% lower than pre-panel (*P* = .02). The slope of change using ITS changed from negative to positive but was nonsignificant (*P* = .26). CAUTI rates per 1,000 PD demonstrated greater reductions (pre- vs post-panel (RR 0.37; 95% CI, 0.29–0.47) and pre- vs post-panel COVID (RR 0.35; 95% CI, 0.26–0.46)). SIRs were unavailable before 2016, but median SIRs post-panel compared to post-panel COVID were similar (1.05 vs 1.56, respectively, *P* = .067).

**Conclusions:**

Implementation of the UTI panel was associated with a reduction in both UC and CAUTI with the impact maintained despite the COVID-19 pandemic.

## Introduction

Urinary tract infections (UTIs) account for 12.9% of all healthcare-associated infections (HAI) in US acute care hospitals with catheter use present in >70%.^1–4^ Catheter-associated urinary tract infections (CAUTI) are associated with mortality, longer hospitalization, and increased detection of antimicrobial-resistant organisms and are viewed by the Centers for Medicare and Medicaid Services as preventable.^5^ Institutions with higher-than-expected HAI rates have been penalized since 2008 increasing focus on their reduction.^2,6^

Interventions to decrease CAUTI have focused on catheter insertion and maintenance as well as decreasing catheter use, including education on indications for catheters, early removal protocols, and bundled order sets.^3,7^ Although these measures have demonstrated some success, facilities have begun focusing on decreasing inappropriate urine cultures (UC) to decrease CAUTI incidence.^8,9^ This is because the National Health Safety Network (NHSN) CAUTI definition is relatively nonspecific, requiring only the presence of a urinary catheter, a fever, and a positive urine culture.^10^ Given that asymptomatic bacteriuria (ASB) is common with indwelling catheters, indiscriminate UC use will lead to potential misidentification of CAUTIs.

To address these issues, we developed a process that integrated clinical documentation and urine microscopy results to determine if a UC was indicated. We removed the ability to order inpatient UC and implemented a UTI panel incorporating evaluating patient characteristics, symptoms, the quality of the urine specimen as judged by the number of squamous cells/low power field (lpf)), and the presence of pyuria to determine if a UC was indicated. We chose lack of pyuria (≤10 WBC/high power field (hpf)) as it has excellent negative predictive value for UTI in immunologically normal patients.^10–12^

The severe acute respiratory syndrome coronavirus 2 pandemic challenged healthcare workers and infrastructure resulting in elevated rates of HAI including CAUTI.^13–15^ Traditional HAI prevention methods were affected by pandemic demands, but we hypothesized that lab-based methods were less likely to be affected. We sought to evaluate CAUTI rates pre- and post-UTI panel intervention, as well as during the COVID-19 pandemic to determine if our intervention led to a reduction in CAUTI rates and if this change was sustained during the COVID-19 pandemic.

## Methods

### Study setting

This study was performed in a 718-bed academic medical center between January 2014 and June 2022. All patients admitted to the hospital during this time were included. We divided the study into 3 periods: pre-UTI panel (pre-panel; January 2014–March 2015), post-UTI panel (post-panel; April 2015–March 2020), and post-UTI panel during the COVID-19 pandemic (post-panel COVID; April 2020–June 2022). Monthly hospitalwide data were evaluated including urinary catheter days (CD), total patient days (PD), UC, and CAUTI count.

### Intervention

**Figure 1.**
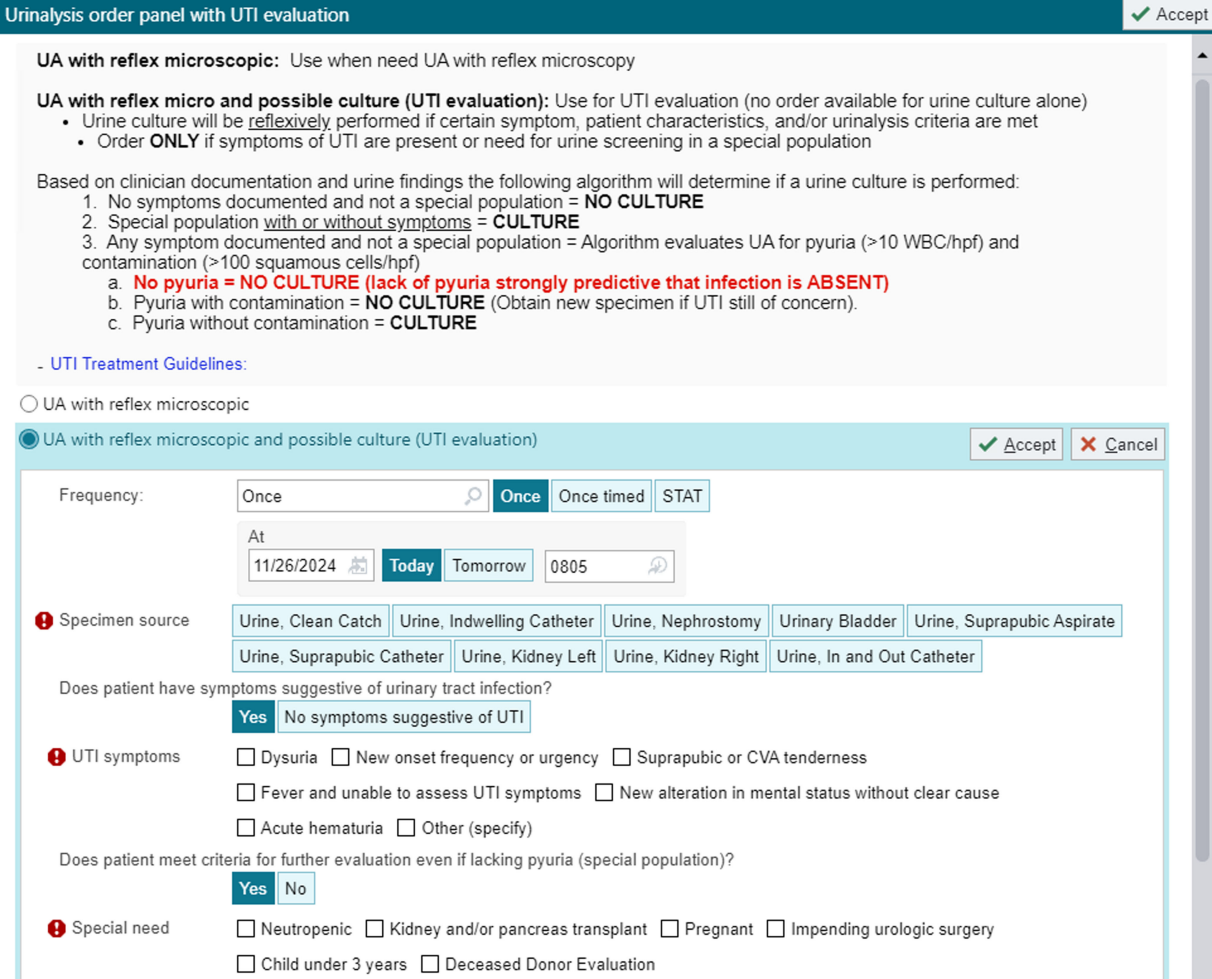
Screenshot of UTI panel in electronic health record ©EPIC 2024.

**Figure 2.**
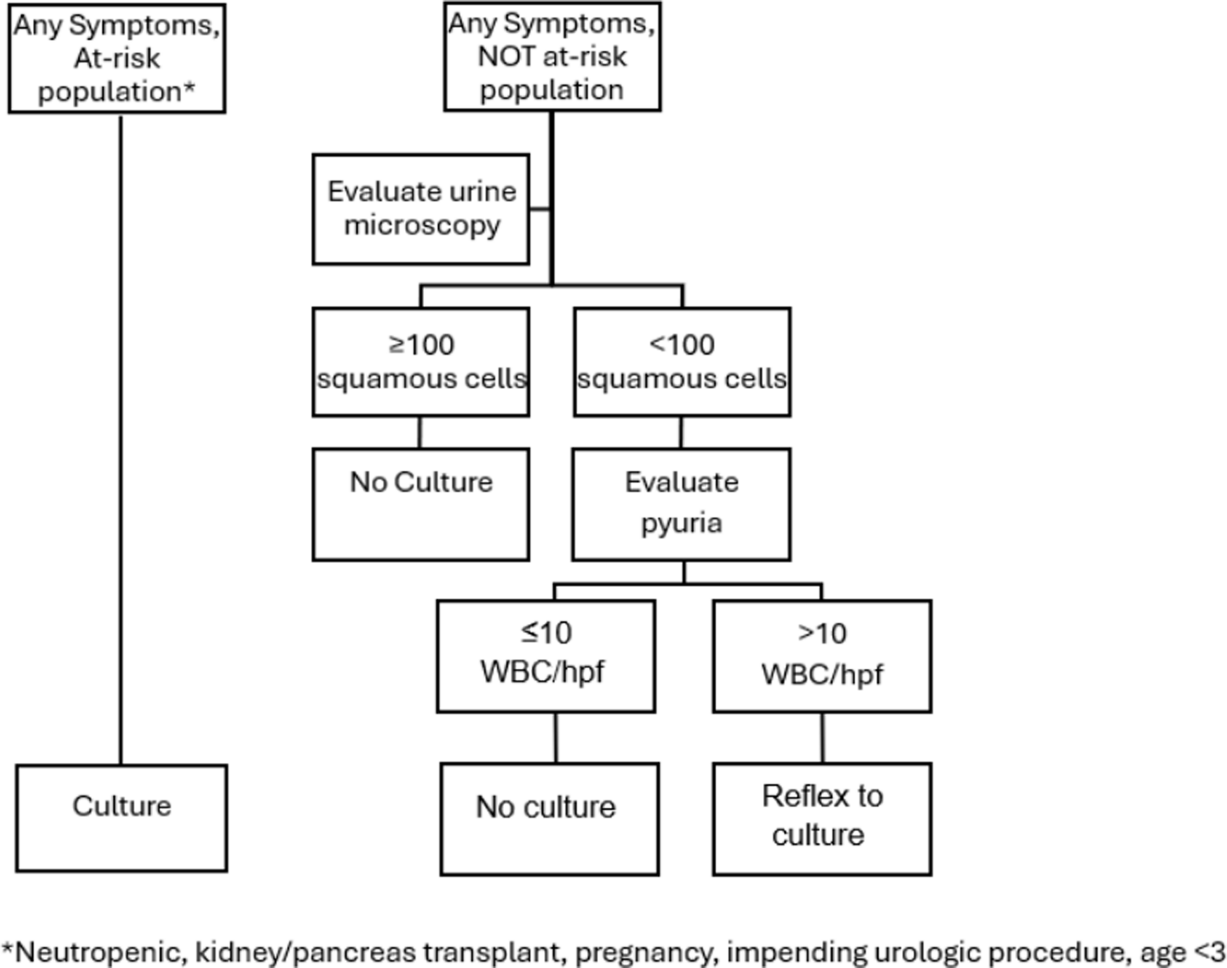
UTI panel algorithm.

In the pre-panel period, urinalysis with reflex microscopy (microscopy performed if leukocyte esterase or nitrites were detected) and a separate UC order were available without restriction or clinical decision support (CDS). UC could be ordered without other urine studies. No reflex UC were performed during this period. In late 2014 and early 2015, a multifaceted CAUTI prevention effort was implemented including required nursing competencies for catheter insertion and maintenance, required indication documentation at catheter insertion, in-depth review of all CAUTI with unit feedback, education on appropriate catheter use and timing of removal, and nurse-driven protocols for catheter maintenance and removal (see Supplemental Material for additional activities). As part of this initiative in April 2015, we removed the ability to order inpatient UC replacing this order with a UTI order panel within our electronic health record (Figure 1). Changes were restricted to the inpatient setting (Emergency Department (ED) and outpatient excluded). An inpatient UC was available only via the UTI panel, and responses were not clinically validated. The panel included an order for urinalysis with reflex microscopy followed by possible UC if specified criteria were met. It required documentation of UTI symptoms and criteria justifying potential UC performance in the absence of symptoms or pyuria. Documentation of the presence of certain conditions resulted in a UC even if no symptoms or urine abnormalities were present (neutropenia, kidney/pancreas transplant, pregnancy, impending urologic surgery, age <3 years, and other with required documentation of the specified condition). When one of these criteria was documented, a UC was always performed regardless of urinalysis findings. Patients who did not have these criteria documented were evaluated for symptom documentation, including typical and atypical symptoms (Figure 2). If no symptoms were documented, a UC was not performed. If any symptom was documented, urinalysis data were evaluated including the number of squamous cells and pyuria. UC performance was based on the following criteria: ≥100 squamous cells/lpf, any number of WBC = no UC as likely contaminated (message to clinicians recommending new specimen); <100 squamous cells/lpf and ≤10 WBC/hpf = no UC as UTI ruled out by lack of pyuria; and <100 squamous cells/lpf and >10 WBC/hpf = UC performed (Figure 2). When a UC was not performed, clinicians could request an override by paging an ID physician to discuss the indication for the culture.

### Definitions

NHSN CAUTI rate per 1,000 CD was the primary outcome with NHSN definitions used throughout the study although the NHSN definition changed in January 2015 (excluded Candida and UC with <100,000 CFU/ml). To adjust CAUTI rates in 2014 infections were recalculated using 2015 definitions with all subsequent CAUTI utilizing the updated definition. Secondary outcomes including CD and CAUTI rates using CD and PD were calculated to adjust for potential changes in denominator data. Quarterly NHSN standardized infection ratio (SIR) and standardized utilization ratio (SUR) rates were compared where available (2016–June 2022).

### Statistical analysis

Poisson regression was used to model the rate of infections per month as a function of period to compare rate ratios (RR). We tested the null hypothesis that rates were similar between the 3 periods. If the null hypothesis was rejected, pairwise comparisons of the 3 periods were conducted, and *P*-values were adjusted using Tukey’s method. A generalized estimating equations approach was used to fit the interrupted time series (ITS) model. The model included an indicator variable for intervention and a continuous variable for time. The model was used to estimate and compare the slopes of the rates of infection for the 3 periods and whether there was a significant difference in the slope between the 3 periods. Interactions were included in the model and used for assessing significant differences in slopes. Separate estimate statements utilizing the interaction terms were included in the model to compare periods. Model checking for the ITS analysis included checking for overdispersion and autocorrelation. SIR and SUR medians were compared using the Wilcoxon rank sum test.

## Results

**Figure 3.**
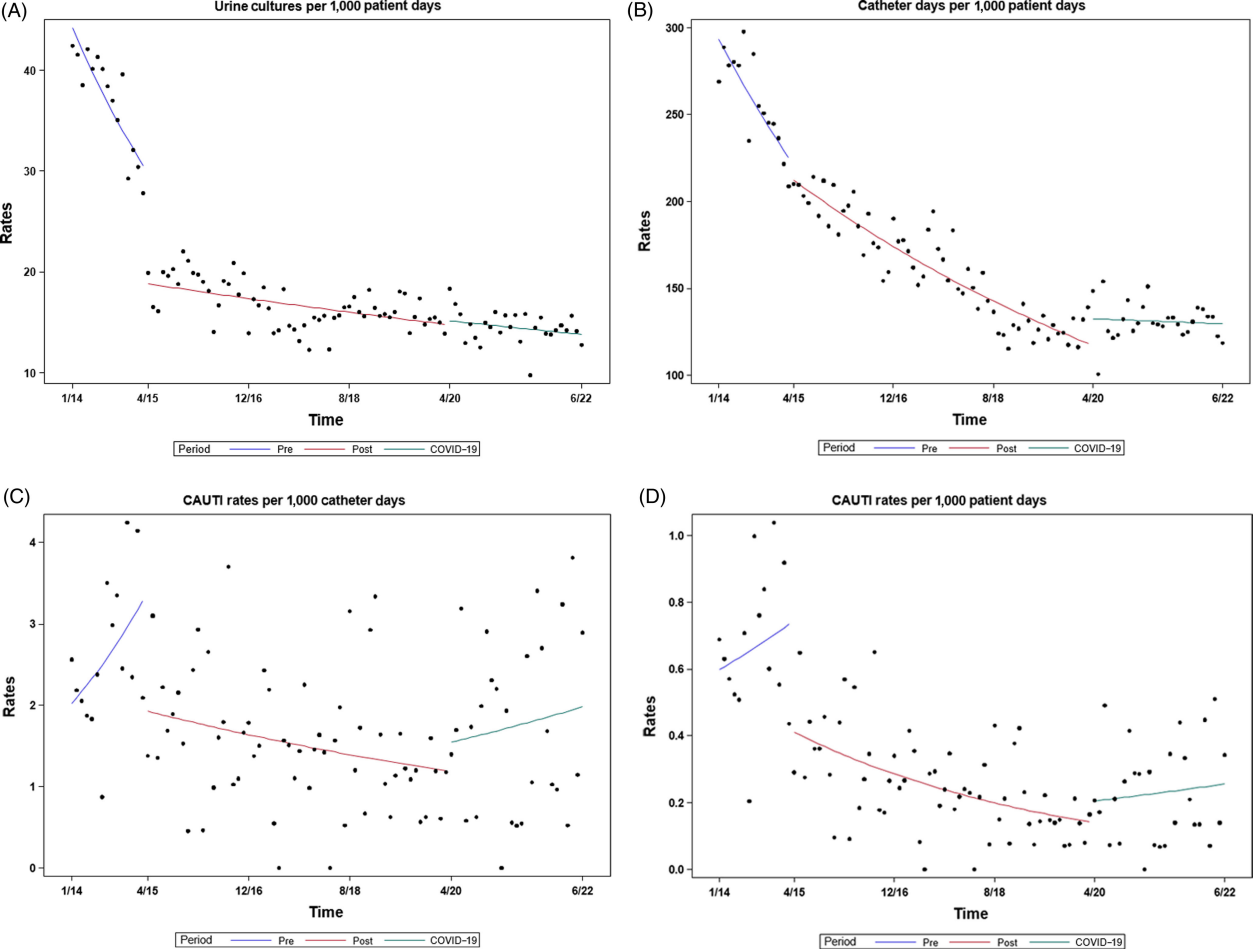
Interrupted time series analysis of monthly data: (A) UC per 1,000 PD. (B) CD per 1,000 PD. (C) CAUTI per 1,000 CD (D) CAUTI per 1,000 PD. UC, urine cultures; PD, patient days; CAUTI, catheter-associated UTI; CD, catheter days.

Throughout the duration of the study, 1,260,713 PD were observed with 204,411 CD. UC were obtained 23,248 times with 368 CAUTI detected. Rates of UC and CAUTI per 1,000 CD and 1,000 PD as well as CD per 1,000 PD are reported in Table 1. Monthly UC, CD, and CAUTI data as well as quarterly SUR and SIR data are present in Supplementary Figure 1. The rate of UC declined throughout the study with both post-panel periods rate decreased compared to pre-panel (Table 1, Figure 3A). There was a 55% decrease in UC comparing pre- to post-panel (RR 0.45; 95% CI, 0.43–0.46; *P* ≤ .001) with an additional 13% decrease in UC between post-panel and post-panel COVID (RR 0.87; 95% CI, 0.84–0.89; *P* ≤ .001) and an overall 61% decrease in UC comparing pre- to post-panel COVID (RR 0.39; 95% CI, 0.37–0.40; *P ≤* .001).

**Table 1.** Change in mean urine culture rates, urinary catheter utilization, and catheter-associated urinary tract infection rates (pre-panel = January 2014–March 2015; post-panel = April 2015–March 2020; post-panel COVID = April 2020–June 2022)

Comparison	Rate comparison	Model estimated risk (95% CI)^a^	*P*
UC/1,000 PD pre-panel vs post-panel	36.90 vs 16.63	0.45 (0.44–0.47)	<0.001
UC/1,000 PD pre-panel vs post-panel COVID	36.90 vs 14.44	0.39 (0.38–0.41)	<0.001
UC/1,000 PD post-panel vs post-panel COVID	16.63 vs 14.44	0.87 (0.84–0.90)	<0.001
CD/1,000 PD pre-panel vs post-panel	257.5 vs 158.1	0.61 (0.61–0.62)	<0.001
CD/1,000 PD pre-panel vs post-panel COVID	257.5 vs 130.9	0.51 (0.50–0.52	<0.001
CD/1,000 PD post-panel vs post-panel COVID	158.1 vs 130.9	0.83 (0.82–0.84)	<0.001
CAUTI/1,000 CD pre-panel vs post-panel	2.59 vs 1.55	0.60 (0.47–0.77)	0.001
CAUTI/1,000 CD pre-panel vs post-panel COVID	2.59 vs 1.77	0.68 (0.51–0.91)	0.02
CAUTI/1,000 CD post-panel vs post-panel COVID	1.55 vs 1.77	1.13 (0.87–1.46)	0.61
CAUTI/1,000 PD pre-panel vs post-panel	0.67 vs 0.25	0.37 (0.29–0.47)	<0.001
CAUTI/1,000 PD pre-panel vs post-panel COVID	0.67 vs 0.23	0.35 (0.26–0.46)	<0.001
CAUTI/1,000 PD post-panel vs post-panel COVID	0.25 vs 0.23	0.94 (0.73–1.21)	0.88

UC, urine cultures; PD, patient days; CAUTI, catheter-associated UTI; CD, catheter days.

a Calculated from the Poisson model comparing later to an earlier period.

CD decreased post-panel compared to pre-panel (Table 1; Figure 3B; rate difference 39%; 95% CI, 0.61–0.62; *P ≤* .001). Cather use continued to decline during the post-panel COVID period although at a lower rate (rate difference 17%; 95% CI, 00.82–0.84; *P ≤* .001).

### Primary outcome

The rate of CAUTI per 1,000 CD was 40% lower post-panel implementation (RR 0.60; 95% CI, 0.47–0.77; *P* < .001) but increased nonsignificantly during the post-panel COVID period (Table 1; Figure 3C; RR 1.13, 95% CI, 0.87–1.46; *P* = .61). Post-panel COVID CAUTI rates were still 32% lower compared to pre-panel rates (RR 0.67; 95% CI, 0.51–0.90; *P* = .02). When CAUTI was evaluated per 1,000 PD greater declines were noted with a 63% decline in CAUTI post-panel compared to pre-panel (Table 1, Figure 3D; RR 0.37; 95% CI, 0.29–0.47; *P* < .0001) with again no change in CAUTI rate post-panel COVID compared to post-panel (RR 0.94; 95% CI, 0.73–1.21; *P* = .88).

### Interrupted time series (ITS) analysis

When CAUTI per CD was evaluated by ITS analysis (Table 2; Figure 3C), there was a significant change from a positive slope pre-panel to a negative post-panel slope (*P* = .01). Although the slope post-panel COVID changed back to positive, this was not a significant change (*P* = .26). A similar pattern was found when CAUTI per PD was analyzed (Table 2; Figure 3D). UC rates had a negative slope in all periods with significant changes in the slope pre-panel to post-panel but not post-panel to post-panel COVID (Table 2; Figure 4A). CD per PD showed a similar pattern to UC.

**Table 2.** Interrupted time series comparison of change in slope over time of urine culture rates, urinary catheter utilization, and catheter-associated urinary tract infection rates (pre-panel = January 2014–March 2015; post-panel = April 2015–March 2020; post-panel COVID = April 2020–June 2022)

Comparison	Slope comparison	*P*
UC/1,000 PD pre-panel vs post-panel	–0.026 vs –0.004	<0.001
UC/1,000 PD pre-panel vs post-panel COVID	–0.026 vs –0.003	<0.001
UC/1,000 PD post-panel vs post-panel COVID	–0.004 vs –0.003	0.85
CD/1,000 PD pre-panel vs post-panel	–0.019 vs –0.010	0.006
CD/1,000 PD pre-panel vs post-panel COVID	–0.019 vs –0.001	<0.001
CD/1,000 PD post-panel vs post-panel COVID	–0.010 vs –0.001	<0.001
CAUTI/1,000 CD pre-panel vs post-panel	0.034 vs –0.008	0.01
CAUTI/1,000 CD pre-panel vs post-panel COVID	0.034 vs 0.010	0.27
CAUTI/1,000 CD post-panel vs post-panel COVID	–0.008 vs 0.010	0.26
CAUTI/1,000 PD pre-panel vs post-panel	0.015 vs –0.018	0.05
CAUTI/1,000 PD pre-panel vs post-panel COVID	0.015 vs 0.009	0.79
CAUTI/1,000 PD post-panel vs post-panel COVID	–0.018 vs 0.009	0.10

UC, urine cultures; PD, patient days; CAUTI, catheter-associated UTI; CD, catheter days.

### Secondary outcomes

Pre-panel SIR and SUR data were not available from NHSN, but data from 2016 to 2020Q1 (post-panel) were compared to 2020Q2–2022Q2 (post-panel COVID). There was a significant difference in the median SUR between post-panel and post-panel COVID periods (0.72 vs 0.56, *P* = .001). The SIR increased nonsignificantly between the 2 periods with a median SIR post-panel of 1.05 versus 1.56 post-panel COVID (*P* = .067).

## Discussion

We demonstrated that the implementation of a UTI panel for reflex UC as part of a multi-component CAUTI reduction project was associated with a rapid and sustained reduction in NHSN-reported CAUTI events. Our UTI panel was unique in that it integrated both clinical and urinalysis parameters. It contributed greatly to the sustained decrease in CAUTI even in the face of decreasing catheter use and COVID-19 pandemic disruptions of traditional CAUTI prevention activities. Our findings suggest that interventions that target UC utilization may be easier to sustain in the face of overwhelming healthcare demands such as those during a pandemic.

To our knowledge, our intervention is the first to combine patient characteristics and symptom documentation with a urine microscopic evaluation to determine the need for a reflex urinary culture. By requiring documentation of symptoms, we sought to improve the predictive value of UC and decrease the detection of ASB. A potential unmeasured benefit may have been a decrease in inappropriate treatment of ASB. With a >60% decrease in UC, we expect a similar decrease in ASB treatment although we did not quantify this potential benefit. Our algorithm also accounted for exceptions to our reflex culturing rules. For example, documentation of the presence of neutropenia always resulted in a UC as the diagnostic performance of pyuria has not been evaluated in this population. Additionally, scenarios, where treatment of ASB is appropriate such as pregnancy and impending urologic surgery, were included as always culture scenarios. The only exception to this rule was if there were >100 squamous cells/lpf when a culture would not be performed as this was highly predictive of a contaminated result. Finally, we allowed clinicians to request ID clinician review for potential overruling in unique circumstances. Developing CDS that anticipates common reasons for algorithm deviation and includes the option for overruling in appropriately reviewed circumstances is important to successful and safe implementation.

We observed decreasing catheter use throughout the study due to ongoing multimodal institutional efforts, which were primarily nurse-driven and maintained throughout the study (see supplemental materials). Because CD are included in the calculation of CAUTI rates, decreases in CD greater than decreases in CAUTI incidence may result in increasing CAUTI rates even when overall infection numbers are stable or decreasing.^16^ Additionally, catheter avoidance is often most challenging in patients at the highest risk for infection, resulting in catheters being removed from relatively low-risk patients and left in place only in those at the highest risk. To account for concomitant changes in catheter utilization, CAUTI rates were calculated using PD, which demonstrated even greater reductions in CAUTI rates although the slope trends were similar.

Based on the immediate and sustained reduction in both UC and CAUTI, we attribute much of our success at decreasing CAUTI to the ability to limit UC. Others have sought to decrease CAUTI by limiting or better utilizing UC with mixed success. One method of altering UC utilization is reflex culturing only when certain criteria are met. Studies suggest that a lack of pyuria in immunologically normal patients has an excellent ability to rule out a positive urine culture although data on ruling out clinical UTI is very limited.^17,18^ Implementing more stringent criteria for reflex UC such as restricting cultures to patients with >10 or >15 WBC/high power field (HPF) can decrease UC from 39% to 57% as well as increase the culture positivity rate.^19,20^ We used a cutoff of >10 WBC/HPF, consistent with expert opinion in immunologically normal patients.^18,21^ It should be noted that before our intervention, inpatient UC were frequently obtained without associated microscopic urine studies as the UC order was not linked to other urine studies. Implementation of the panel required that UC be linked to a urinalysis, allowing for both reflex culturing and providing clinical data for results interpretation.

There has been an increasing focus on improving the appropriateness of UC use to decrease CAUTI. Virtually all studies have decreased UC rates, but the impact on CAUTI has been mixed. Two studies that used CDS tools to provide education on appropriate UC use decreased UC but did not decrease CAUTI.^22,23^ Another strategy is gatekeeping of UC where cultures cannot be obtained without certain criteria being met or the test being approved by a clinician. For example, Mena Lora et al educated providers on UC utilization and required ID approval of any UC performed in a patient with a urinary catheter resulting in a >50% decrease in UC and even greater reductions in CAUTI.^24^ Another site used an interruptive alert to successfully decrease CAUTI by requiring justification of UC performance in patients with urinary catheters with non-appropriate indications resulting in no UC.^16^ Education has been used successfully to decrease UC and CAUTI although success has usually been associated with either carefully targeted groups (Intensive Care (ICU) only), repeated education, or education coupled with feedback on performance.^9,25^ Our UTI panel combined multiple elements including education, CDS, reflex culturing, and some components of gatekeeping, which is why we hypothesize it was and remained highly successful.^20,26,27^

One concern when implementing diagnostic stewardship interventions is the potential for missed opportunities to obtain a diagnosis due to more stringent testing. Although we did not evaluate the effect of our intervention on adverse events such as bacteremia, previous studies have demonstrated decreased UC without an observed increase in gram-negative bloodstream infections or hospital or UTI mortality.^9,28^

Our study also included the COVID-19 pandemic period, which was a singularly challenging time for HAI prevention. Weiner-Lastinger et al reported a 36% increase in the number of CAUTIs reported to NHSN in 2020 compared to 2019.^14^ Evaluation of CAUTIs in 1 US hospital network noted a 43% increase in CAUTI compared to the pre-pandemic rate.^29^ At our center, the slope of CAUTI per CD did switch from negative to positive, and the number of CAUTI did increase although both changes were not statistically significant. Similarly, a nonsignificant SIR increase was noted in the post-panel COVID period. Comparing post-panel to post-panel COVID periods, the mean CAUTI cases increased from 3 post-panel to 3.26, and the CAUTI rate increased by 13%, but when measured by PD, it declined by 6%. The SIR change was greater proportionally than the increase in infections. Many factors may have contributed to this difference during COVID including differences in patient severity of illness, comorbidity, or length of stay. Changes in catheter use or HAI surveillance due to the COVID burden might have influenced the SIR. Finally, SIR calculations adjust for unit type, and numerous unit designation changes occurred at our facility during the post-panel COVID period.

Our study has potential limitations. First, the study was conducted in a single academic center although the intervention could be potentially implemented in most facilities. Lack of patient-level comorbidity data may affect generalizability as our center includes large oncology and solid organ transplant programs. However, as this intervention was utilized in these patients, it suggests it may be effective in diverse patient populations. Outcomes other than CAUTI were not tracked, and therefore, we are unable to quantify changes in other events such as non-CAUTI UTI, gram-negative bloodstream infections secondary to UTIs, antibiotic side effects, and the development of antimicrobial resistance related to the treatment of ASB or CAUTI. The study was quasi-experimental with the UTI panel implemented as part of a multimodal CAUTI prevention program that evolved over time. Thus, it is likely some of the impact on CAUTI was from the numerous other interventions. The major change noted in the CAUTI trajectory with the implementation of the UTI panel suggests it was the primary factor influencing CAUTI rates. We did not validate that the panel was used correctly, and clinicians may have used the special category to obtain a UC inappropriately. Neither did we monitor the rate of ID-approved overruling although one of the authors is the primary person responsible for this (TCV), and he noted this occurs less than once per month. We did not evaluate unit-specific data, so we are unable to assess if there were different impacts in different units. We did not evaluate the potential benefit of decreased ASB detection and treatment.

## Conclusion

Implementing an intervention combining clinical data with reflexive culturing based on urinalysis findings as a component of a multimodality CAUTI prevention program resulted in a major decrease in both the number of UC performed and CAUTI detected. Although the specific contribution of the UTI panel is not fully defined, the data we present suggest it was the major driver of the decrease in CAUTI rates. These changes were sustained during the COVID-19 pandemic, demonstrating the benefits of lab-based interventions that do not require ongoing personnel time or maintenance efforts.

## Supporting information

Torres et al. supplementary material Torres et al. supplementary material

## Supplementary material

The supplementary material for this article can be found at https://doi.org/10.1017/ice.2025.2.
