# Supplementary material for: Dropping the urine culture: sustained CAUTI reduction using a UTI order panel
Source: Infect Control Hosp Epidemiol. 2025 Feb 13;46(4):377–83. doi: 10.1017/ice.2025.2 (PMC12015623; doi:10.1017/ice.2025.2)
Supplement: Torres et al. supplementary material [file S0899823X25000029sup001.docx]

**Supplemental Information on CAUTI Reduction Activities 2015-2022**

2015

- Indwelling Urinary Catheter (IUC) Removal Protocol created with clinician education
- Infection preventionist rounding on unit to assess foley need and maintenance
- Urinary Management Protocol directing use of non-indwelling catheter created
- Indwelling Urinary Catheter Protocol instituted defining maintenance of catheter

2018

- CAUTI Deep dive tool created to assess potential factors resulting in CAUTI with feedback of findings to unit leadership

2019

- Infection Control Department performed assessment of CAUTI prevention using CDC CAUTI Toolkit (Core Prevention Strategies/Supplemental Prevention Strategies), CAUTI Deep Dive data FY2018, and CAUTI ICE data.
- Findings of assessment including strengths and opportunities presented at Nurse Director/Manager meeting
- Hands on Foley Insertion competencies/Foley Cares competencies instituted house wide for nursing staff

2021

- CAUTI/CLABSI Steering committee formed to direct HAI prevention efforts

2022

- CAUTI Task Force completed assessment of CAUTI Prevention using CDC CAUTI Toolkit (Core Prevention Strategies/Supplemental Prevention Strategies) and other Infection Control data
- CAUTI Task Force prioritized opportunities using a Control vs Impact grid
- CAUTI Task Force updated CAUTI Deep Dive tool
- Trialed and adopted new external male catheter product
- Traveling nurse orientation class initiated including CAUTI prevention education
- Hands on Foley Insertion competencies/Foley Cares competencies began again house wide for nursing staff
- EHR update to nursing worklist task trigging foley cares due and documentation reminder
- CAUTI Maintenance Bundle charting instituted

**Supplemental Figure 1.** Monthly or Quarterly Changes in Study Parameter: A) Urine Cultures per 1000 PD B) Catheter Days per 1000 PD C)-CAUTI per 1000 Catheter Days D) CAUTI per 1000 PD E) Quarterly NHSN Urinary Catheter Standardized Utilization Ratio (SUR) F)- Quarterly NHSN CAUTI Standardized Infection Ratio (SIR).


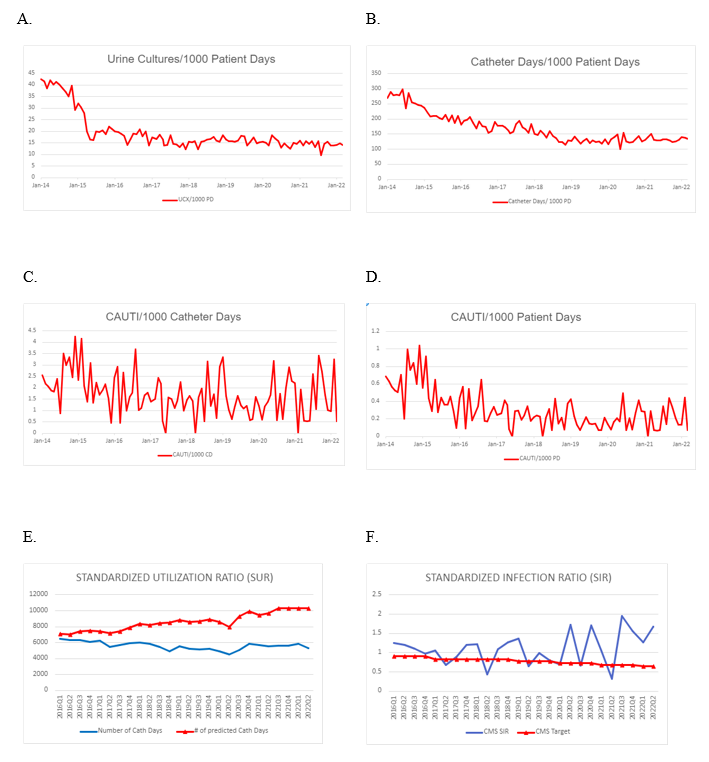


PD=Patient days; CAUTI=Catheter-associated UTI; CD=Catheter days
